# Markers for Detection of Prostate Cancer 

**DOI:** 10.3390/cancers2021125

**Published:** 2010-06-04

**Authors:** Raymond A. Clarke, Horst J. Schirra, James W. Catto, Martin F. Lavin, Robert A. Gardiner

**Affiliations:** 1 Prostate Cancer Institute, Cancer Care Centre, St George Hospital Clinical School of Medicine, University of New South Wales, Kogarah, NSW 2217, Australia; E-Mail: r.clarke@unsw.edu.au; 2 School of Chemistry and Molecular Biosciences, University of Queensland, Brisbane QLD, 4072, Australia; E-Mail: h.schirra@uq.edu.au; 3 Academic Urology Unit and Institute for Cancer Studies, University of Sheffield, Royal Hallamshire Hospital, Sheffield S10 2JF, UK; E-Mail: j.catto@sheffield.ac.uk; 4 Queensland Institute of Medical Research, Radiation Biology and Oncology, Brisbane, QLD 4029, Australia; E-Mail: martin.lavin@qimr.edu.au; 5 University of Queensland Centre for Clinical Research, Brisbane, Australia

**Keywords:** prostate cancer, biomarker, detection

## Abstract

Early detection of prostate cancer is problematic, not just because of uncertainly whether a diagnosis will benefit an individual patient, but also as a result of the imprecise and invasive nature of establishing a diagnosis by biopsy. Despite its low sensitivity and specificity for identifying patients harbouring prostate cancer, serum prostate specific antigen (PSA) has become established as the most reliable and widely-used diagnostic marker for this condition. In its wake, many other markers have been described and evaluated. This review focuses on the supporting evidence for the most prominent of these for detection and also for predicting outcome in prostate cancer.

## 1. Introduction

Prostate cancer (PCa) is now the most commonly diagnosed internal cancer in the western world and the second most lethal male malignancy. Prior to clinical availability of a blood test for prostate specific antigen (PSA) in the mid 1980s, most PCa patients were diagnosed with more advanced disease. 

Advances in surgical and radiation therapies now result in cure for a proportion of patients who would not have benefitted from combination PSA/digital rectal examination (DRE)-based detection in the past. However, from a public health perspective, indiscriminate PSA population screening cannot be justified because of the low sensitivity and specificity of this blood test in identifying cancer, amongst other concerns [1]. Furthermore, there is prevailing uncertainty on the basis of the PSA blood test as to who will benefit from treatment with curative intent [2,3,4,5]. Recognition of these shortcomings has focussed research into markers to detect the presence of PCa and, more importantly, to try to discriminate between those patients harbouring indolent as opposed to aggressive disease including those for whom any form of local therapy will not prevent subsequent declaration of metastases.

There is a clinical imperative in being able to identify those who do and do not have PCa since, apart from the obvious advantage from detecting and treating the condition at the earliest stage possible, the diagnostic process itself is not without its problems. Suspicion of the presence of PCa, alerted as a result of an abnormal serum PSA and/or and abnormal DRE, requires an invasive procedure to provide histological confirmation with biopsies (usually >12) almost always obtained via imaging with a transrectal ultrasound (TRUS) probe to ensure spatial positioning of biopsy needles.

The majority of men who have prostatic biopsies do not have PCa diagnosed but continue to have raised serum PSA levels with ongoing concerns of a possible undetected PCa due to the imprecise nature of both the PSA test and TRUS biopsies. US Medicare-SEER analyses 1993 to 2001 indicated that, among men whose first recorded biopsy did not detect cancer, the likelihood of undergoing subsequent biopsies was 11.6% at 1 year and 38% at 5 years [6]. In addition, although the dreaded complication of life-threatening sepsis is fortunately uncommon but reported to be increasing [7], ~50% experience lesser symptoms after TRUS biopsy so diagnosing PCa remains problematic for many reasons. Consequently, it is not surprising that the need to be able to detect prostate cancer by a simple and reliable approach which can be repeated easily over time, is a diagnostic priority in the western world.

## 2. Scope of the Review

The strengths and weaknesses of PSA in the detection of PCa have been examined and published extensively and, since the topic has been reviewed very recently by Roobol *et al.* [8], who comprehensively examined the relevant medical literature with respect to the risk of developing PCa, this dissertation will not re-visit this marker. These authors reported that, despite its limitations, published evidence indicates that total serum PSA is the single most significant clinically-used predictive factor for identifying men at increased risk for PCa. Total PSA was superior to percentage free PSA, PSA velocity and Human kallikrein 2 (hK2), the most studied kallikrein protein after PSA (hK3) itself. A suspicious DRE, a family history of PCa, the presence of high grade prostatic intraepithelial neoplasia (HGPIN) or atypical small acinar proliferation (ASAP) and black ethnicity also were reported to be important predictive factors. For men of screening age (50 to 70 years) a serum PSA of greater than 1.5 ng/mL has been found to indicate a greater than average risk up to 8 years (7.5-times greater risk *vs.* 1.5 ng/mL or less) for developing PCa [8]. In terms of diagnosing established PCa, PSA cut-offs are useful with the likelihood of men harbouring PCa greater the higher the level of serum PSA. However, it is important to note that PSA is a continuous variable and that a proportion of patients with low serum PSA levels <4 ng/mL have PCa of clinical significance [9]. 

Histological interpretation too has advanced with the Gleason scoring criteria agreed upon at the WHO ISUP consensus meeting in 2005 [10,11] being more useful clinically than those criteria with Gleason grading used previously. Much has been published in the Uro-pathology literature in relation to histological nuances in addition to describing a range of tissue biomarkers for PCa characterisation. These include apoptotic factors such as p53 and Bcl-2, the androgen receptor (AR), signal transduction factors within the EGF receptor family, cell cycle regulators exemplified by c-Myc, p16, p27, pRb and Ki67, cell adhesion and cohesion factors and factors involved in neo-angiogenesis, such as vascular endothelial growth factor (VEGF), VEGF receptors and nitric oxide [12,13] 

Histopathology will not be addressed further nor will imaging adjuncts to biopsy. However, in interpreting the value of diagnostic biomarkers from biological fluids, which is the subject of this review, accurate histology and appropriate targeting with biopsy needles are essential. It is particularly important to be aware that the diagnostic biopsy reference on which biological fluid biomarkers are judged is deficient, varying with sites targeted and numbers of biopsies obtained, ranging in one study from 31.7% for sextant biopsies to 38.7%, 41.5%, and 42.5% for 12, 18 and 21 cores respectively in a study of 1000 patients [14]. 

Accurately determining the presence of disease is surprisingly difficult. Autopsy studies permit exhaustive and comprehensive scrutiny of the prostate histologically but are flawed in terms of clinical relevance for epidemiological reasons. True incidence derived from more epidemiologically-vetted clinical trials is almost non-existent given the rarity of complete cross-sectional ‘end of study biopsies’. Sakr found in autopsy studies that, for the third through to the eighth decades of life, the incidence of PCa in a cohort of 1051 subjects was 7, 23, 39, 44, 65 and 72% [15]. 

Haas *et al.* [16] performed 18 core biopsies on autopsy prostates from 164 men who had no history of PCa. They reported that six-core biopsies were taken from each of the mid peripheral zone (MPZ), the lateral peripheral zone (LPZ), and the central zone (CZ). PCa was present in 47 (29%) prostates. Of the 47 cancers detected, 20 were clinically significant according to histologic criteria. Biopsies from the CZ did not detect any cancer that was not present in biopsies from either the MPZ or LPZ. The sensitivities of biopsies from the MPZ for clinically significant and insignificant cancer were 55% (95% CI = 32% to 77%) and 11%, respectively, compared with 80% and 33% for those from the MPZ and LPZ combined. In a single institutional study, Djavan *et al.* [17] found in an assessment of repeat biopsies that PCa was present in 22% (231/1051), 10% (83/820), 5% (36/737) and 5% (4/94) of first, second, third and fourth biopsies. Together with others, these studies illustrate the fact that the reference by which the sensitivity and specificity of biomarkers are judged is well short of perfect and this deficiency also should be borne in mind in assessing the predictive values of potential biomarkers.

## 3. Sources for Biomarker Analyses

A number of tissue/fluid sources and approaches have been employed in the quest to develop a non-invasive diagnostic test for PCa. These range from analysing tears [18] to changes in the odour of urine detected by dogs [19] but, in terms of commonly studied sources, prostatic fluid and blood have received most attention. Patient acceptance of venesection for taking samples of blood (for analysis as whole blood or serum) and providing urine is extremely high and laboratory staff are so conversant with the properties of both that they do not warrant detailed discussion in this manuscript.

However, the use of prostatic fluid is not nearly as well appreciated. It is logical that fluid and/or cells obtained as directly as possible from the PCa itself should have differences in greatest abundance to those from normal prostates. Prostatic fluid may be obtained either as post-prostatic massage urine or ejaculate and/or post-ejaculate urine. 

### 3.1. Post-Prostatic Massage Urine

Since DRE by itself dislodges too few retrievable prostatic cells, most studies using prostatic fluid have obtained specimens for analysis by prostatic massage [20,21,22,23,24,25,26,27,28,29] which is recommended to involve 3 sweeps per lobe, optimally depressing the prostate by 0.5–1 cm in a milking action to ensure sufficient numbers of prostatic cells are obtained [30]. Micturition following massage then washes prostatic fluid and cells dislodged into the prostatic urethra to the external meatus for collection.

**Figure 1:**
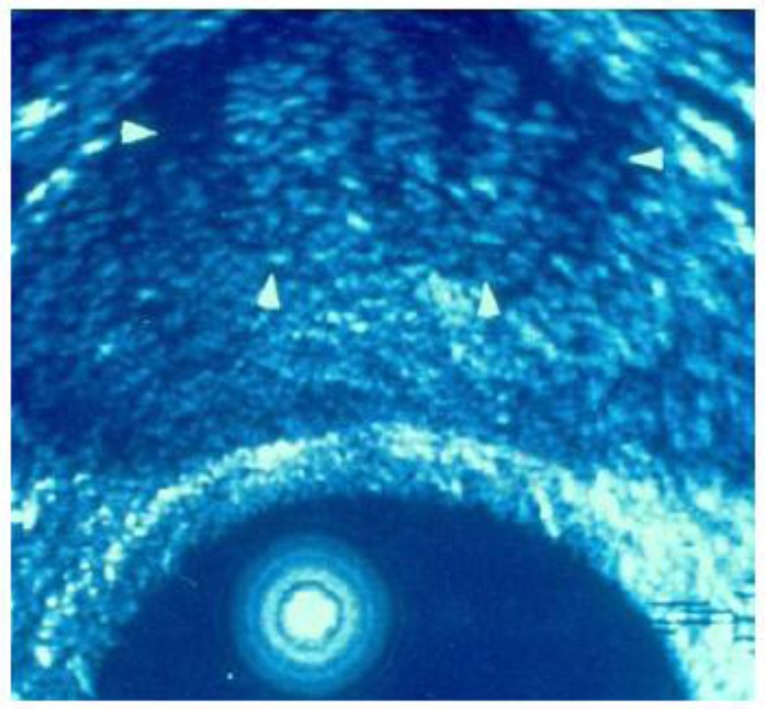
Ultrasound probe in rectum illustrating the relative relationship of rectum and prostate: white arrows indicate the transition zone (TZ).

Although there is a low likelihood of testicular cells or sperm being collected with this approach, one limitation is that only the MPZ is directly targeted for massaging with the anterior LPZ missed completely. In addition to being unpleasant, a further potential problem with vigorous prostatic massage is dislodging cancer cells into the systemic circulation with possible adverse consequences in terms of facilitating metastatic spread although evidence at 5 years does not support accelerated cancer evolution judged as a result of blood seeding cancer cells during operative intervention [31].Figure 1

### 3.2. Ejaculate

Prostatic fluid with disaggregated cells in ejaculate is expelled during orgasm following contraction of prostatic smooth muscle, distributed widely throughout the gland. In addition to the prostatic component, ejaculate contains contributions from the testes, epididymi and seminal vesicles and there is a tendency for a reduction in ejaculate volume with ageing. Certainly, following resection of the bladder neck with transurethral resection of prostate (TURP), there is retrograde emission so that seminal fluid mixes with urine to be expelled subsequently during micturition. 

Much has been made of a reduced capability of older men to be able to perform but evidence in the medical literature [32,33] is to the contrary. Our experience has been that when the request is optional and not directly related to management outcome, a proportion of patients, although capable, will elect not to comply in producing a specimen in private and returning it promptly to the laboratory but, if there is a diagnostic imperative such as a sperm analysis, the response is quite different. Therefore, using ejaculate as a preferred body fluid for PCa testing is considered a practical option.

Impotence does not exclude an ability to ejaculate although firm tumescence certainly helps with this process. It is notable that erectile dysfunction is now well-recognized as a sentinel event for underlying cardiovascular disease and has been reported to be a predictor for the combined outcome of acute myocardial infarction, stroke and sudden death [34]. Therefore, an inability to ejaculate may translate as a surrogate for identifying a subset of patients for whom a diagnosis of PCa is less likely to affect life expectancy in the context of what is a relatively slowly progressing malignancy in the majority of cases. 

## 4. Identifying Discriminating Markers

Although the cause of PCa continues to remain elusive, family histories indicate a strong inherited genetic predisposition in both the risk of developing PCa and the likelihood of a poor outcome should PCa be present. Not only is a family history of PCa with early age of onset and numbers of first degree relatives especially relevant in identifying predisposition [35], but a family history of breast cancer is also emerging as important especially in relation to BRCA2 mutations. Certainly, BRCA2 mutations are reported to be uncommon in sporadic PCa, estimated as <1% in US patients and 2.3% in UK patients [36] and also not common in hereditary PCa [37]. However, men who do develop PCa and harbour a BRACA2 mutation are more likely to have aggressive tumours and tend to develop these malignancies at an earlier age than other patients [36,38]. 

Genome-wide association studies (GWAS) indicate genetic heterogeneity for the onset of disease with numerous low risk loci described along with two notable high-risk loci at 8q24 and 7q31. The linked loci on 8q24 are located immediately downstream of the MYC gene that is upregulated in PCa [39,40] and linkage at 7q31 in African American men is in close proximity to the PODXL gene [41] which has been mutated in men with aggressive forms of the disease [42]. It is noteworthy that a combination of a family history of PCa together with genetic SNP variations increases the risk association significantly. In addition to genetic heterogeneity there is also a high likelihood that PCa genes/alleles act cooperatively in the aetiopathogenesis of the disease supporting the notion that it is unlikely that any one biomarker alone is likely to be conclusive in detecting and predicting outcome. The Practical consortium recently reported the conclusion of their successive GWAS studies. They evaluated over 500,000 alleles (SNPs) in 3 separate cohorts of cases/controls (up to 30,000 men [43]. The consortium eventually identified alleles in 7 genomic regions (encompassing 11 genes) that were linked to PCa diagnosis. The allele penetrance ranged from 6–50% of the population and each allele altered PCa risk by 0.35–1.89 (odds ratio).

Chromosomal aberrations are very common in prostate tumours [44] and may act together in the progression of the disease. For example, the loss of both the PTEN gene on chromosome 10q and a recurrent 30Mb deletion on 21q, that leads to the fusion of TMPRSS2 and ERG (see section on ETS fusions), are common aberrations which appear to act together in disease progression [45]. Gain and amplification of the MYC gene on 8q24 [44,46] is of great interest especially given the strong GWAS linkage established immediately downstream of the gene [39,40]. 

## 5. Assay Approaches & Most Promising Markers

In reaching the current position with respect to identifying markers of detection, RNA profiling using microarray-based technologies has been particularly useful in tracking changes in gene expression during tumorigenesis. To date, the two most prominent candidate RNA biomarkers are the PCA3 gene and TMPRSS2 fusion transcripts. These, together with other genetic and non-genetic markers, are discussed below with respect to their likelihood in differentiating cancer from non-cancer and identifying indolent as opposed to aggressive and life-threatening tumours.

Given the many markers reported in the scientific literature, we have had to follow the practice employed in other reviews of selecting a short list of those which are considered to be most relevant and most promising.

### 5.1. Markers of Detection *versus* Prognosis

Arguably far more important than a diagnosis of PCa is the true nature of the condition in an individual patient. Although, without doubt, timely intervention does save lives, it is indisputable that many men who have treatment with curative intent for PCa are unlikely to benefit in terms of survival [47,48,49]. However, all these patients are at-risk of not inconsiderable side effects from intervention including previously unrecognised problems afflicting both them and, indirectly, their loved ones [50,51,52,53]. Contemporarily, the large majority of patients are diagnosed with PCa as a result of PSA testing and ~30% are stratified to low-risk disease with another ~25% already having occult metastases which declare themselves subsequently [54,55]. Because of the disparity between TRUS biopsy findings and those of whole-gland histology, there is a tendency for many urologists and patients to err on the side of intervention and to proceed to treatment with curative intent, accepting that a significant proportion of patients will have treatment that will not affect survival or benefit their well-being.

In order to identify the minority of patients stratified as having low-risk PCa following biopsy but whose disease is likely to progress, Klotz initiated an active surveillance protocol [55] undertaking a period of very intense 2-year monitoring of these men with PSA testing, DRE and at least yearly TRUS biopsies to unearth the ~25% of men who actually have more aggressive disease not evident from initial TRUS biopsies [55]. This approach has been emulated by colleagues in the UK, US and Europe and a randomised study has been undertaken to examine this strategy further, in the Surveillance Therapy Against Radical Treatment (START) trial. Klotz has the largest and most mature study of active surveillance and has reported an 85% overall survival and 99% disease specific survival with a median follow-up of 8 years (range 2–11 years) [55]. Clearly there is an overwhelming need for a prognostic profile that would, non-invasively, permit accurate stratifying of PCa patients.

A distinction between detection, prediction and prognosis is not clear for many of the markers reported with some having the potential to fulfil both roles. Identification of potential markers has been based largely on histological expression of candidates but, interestingly, it is common for a disparity to exist between tissue expression and detection in various biofluids [56]. Furthermore, although many of the biomarkers identified have been at a protein level or produce protein(s)/peptides, there are many such as microRNAs, non-coding RNAs and metabolic products not directly associated with proteins or peptides contributing to the pot-pourri of potentially useful biomarkers for identifying the presence and nature of PCa non-invasively.

## 6. Comments on Selected Genetic Markers

A selection of biomarkers is listed in Table 1 & Table 2 (below). For the multivariate analysis, AIC-based backward selection was used to drop insignificant terms.

**Table 1:** Post-prostate massage-urine biomarkers for the detection of prostate cancer from Laxman *et al.* [26].

Variable	Coefficient	*P*
Univariate logistic regression analysis		
*GOLPH2*	0.4444	0.0002
*SPINK1*	0.25	0.0002
*PCA3*	0.187	0.001
*TMPRSS2:ERG*	0.609	0.034
*ERG*	0.043	0.166
*TFF3*	0.11	0.189
PSA (serum)	0.0151	0.376
*AMACR*	0.049	0.45
Multivariate logistic regression analysis		
***SPINK1***	0.308	7.41E-05
***PCA3***	0.191	0.003
***GOLPH2***	0.372	0.004
***TMPRSS2:ERG***	0.924	0.006

**Table 2:** Potential urine markers for monitoring prostate cancer, modified from Jamaspishvili *et al.* [57].

Symbol	Description	Type of marker				Ref.	Body Fluid
		DNA	RNA	Protein	Metabolite		
8-OhdG	8- HydroxydeoxyguanosineU	+			+	[58]	U
ANXA3	Annexin A3			+		[59,60,61,62]	PD
BHUAE	Basic human urinary arginine amidase			+		[63]	U
F3	Coagulation factor III (thromboplastin, tissue factor)			+		[64]	U
GSTP1	Glutathione S-transferase P 1	+				[65,66,67,68,69]	PM
**LOH **	Loss of heterozygosity e.g., loss of PTEN	+				[70,71]	PM
MCM5	Minichromosome maintenance complex component 5			+		[72]	U
MMP9	Matrix metalloproteinases 9			+		[73,74,75]	U
PIP	Prostatic inhibin-like peptide			+		[76]	U
PSA	Urinary prostate specific antigen			+		[77]	U
S100A9	S100 calcium binding protein A9 (alias calgranulin B)			+		[78]	PM
**SAR **	Sarcosine				+	[79]	PD
SRD5A2	Steroid 5-alpha-reductase type 2			+		[80]	U
TERT	Telomerase reverse transcriptase		+			[81,82,83]	PM
TMSB15A	Thymosin beta 15a			+		[84]	U
**VEGF**	Vascular endothelial growth factor			+		[85,86]	U

Key: PM~ post-prostate-massage urine, PD ~ post digital examination urine, U~ voided urine.

### 6.1. PCA3

PCA3, previously known as *DD-3*, is a non-coding gene first reported by Bussemakers *et al.* [87]. mRNA of the PCA3 gene is highly over-expressed (median 66-fold) in >95% of PCa tissue compared with normal or benign prostatic tissue of the same patients [20,87]. PCA3 has been assayed from urine following prostatic massage in 11 separate clinical studies totalling 2737 men from Western countries [21,22,23,24,28,30,88,89,90,91,92,93] with an overall sensitivity of 69% and specificity of 70% for men with PCa.

The role of PCA3 in clinical practice as a commercially-available test remains uncertain with most advocates indicating a place in patients who have already had TRUS biopsies with a negative result for cancer but in whom PCa remains suspected. Another application may be as an adjunct to repeat DREs, serum PSA estimations and prostatic biopsies in following men diagnosed with low-risk PCa who have elected to be monitored carefully in active surveillance protocols rather than have treatment with curative intent. However, more recently, a number of investigators have combined their PCA3 findings with those from other markers to try to incrementally improve detection rates since it is clear that no one marker by itself is adequate for detecting all cases of PCa.

### 6.2. PCA3 Redefined

**Figure 2:**
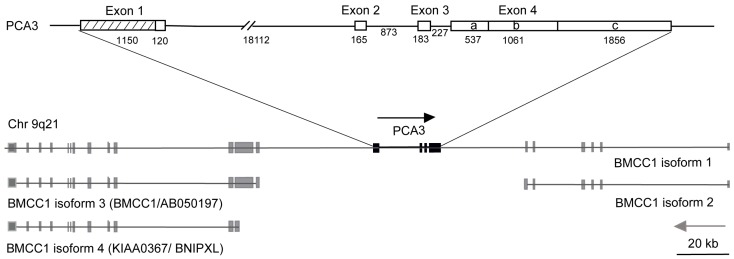
Redefined PCA3 gene.

Although upregulation of *PCA3* was first described in PCa specimens in 1999 [87], it was another 10 years before the complete structure of the gene was resolved [94] with description of new start sites, additional exons and a range of novel alternatively-spliced transcripts (Figure 2), some of which are more highly enriched in PCa and metastases, expression not normally being seen outside the prostate. *PCA3* is embedded in an anti-sense orientation within an intron of another much larger gene, *BMCC1-1*. In contrast to *PCA3*, a weakly conserved non-coding gene, *BMCC1-1* has a conserved protein domain with an established role in Rho-signalling, cellular transformation and metastasis [94,95].

*PCA3* expression is androgen responsive with alternatively spliced *PCA3* mRNA transcripts differentially upregulated in up to 95% of PCas and metastases [94,95,96]. *PCA3* mRNA levels have been detected in primary and metastatic PCa tissue specimens at up to 43 and 110 times the levels expressed in normal prostate tissue, respectively, compared with up to a 3 fold increase in benign prostatic hyperplasia (BPH) [94]. By combining *PCA3* with 2 other biomarkers (*i.e.*, *PSMA* and *hepsin*) 100% of tissue samples (55 in total) were classified as either PCa or BPH [97]. The recent discovery of novel *PCA3* transcripts, inclusive of exons 2a and 2b, that are more highly enriched in PCa and metastases and the identification of complementary biomarkers [26,97] tantalises investigators who seek further improvements in PCa detection and characterisation through a non-invasive molecular approach.

*PCA3* is expressed at high levels in metastatic PCa but, empirical data do not support an association between *PCA3* upregulation and clinical stage, Gleason scores, tumour volume, pathological stage or cancer progression [93,98]. Nevertheless, the genomic inter-relationship between *PCA3* and the different *BMCC1* isoforms [94] may not be coincidental and investigations are underway for clues to the association between *BMCC1* regulation and PCa initiation and progression [94].

The truncated form of *PCA3* described by Bussemakers *et al.* [87] was extended by Clarke *et al.* [94] using a thorough 5’ and 3’ RACE analysis of the mature message and DNA and RT-PCR sequencing in their description of the complete *PCA3* gene with:

- Exon 1 over 10 times longer than previously reported
- 4 new transcription start sites
- 4 polyadenylation sites
- 2 new differentially spliced exons
- PCA3 embedded in intron 6 of the *BMCC1-1* gene

### 6.3. ETS Gene Fusions

ETS gene fusion provides a highly specific biomarker assay for the detection and prognosis of PCa. In 2005, microarray analyses identified two E26 avian erythroblastosis virus (ETS) family transcription factors, ETS related gene (ERG) and ETS variant gene 1 (ETV1), as potentially important PCa biomarkers [99]. Aberrant expression of these ETS genes was found coincident with gene fusion between the 5′ untranslated region of androgen-regulated transmembrane protease, serine 2 (TMPRSS2) gene and the ETS genes *ERG* (chromosome 21q22.2), *ETV1* (7p21.2), *ETV4* (17q21) or *ETV5* (3q28) or one of ~20 other gene fusion variants [100,101,102,103]. ERG and *ETV1* rearrangement and overexpression has been reported to occur in the majority (50–60%) of PCas with the most common variant being a recombination between exon 1 of *TMPRSS2* and exon 4 of *ERG*, designated T1/E4 [104,105,106,107,108] represented in ~85% of all reported fusion cases [101,109]. Identification of ETS fusions in the ‘post-prostatic massage urines’ from men with PCa using RT-PCR has a sensitivity of 37%, a specificity of 93%, a negative predictive value of 36% and positive predictive value of 94% [25,110,111].

ETS gene fusions have an important role in PCa development and prognosis. *ERG* and *ETV1* show mutually exclusive overexpression in PCa tissues, suggesting a redundant function in PCa development. In addition, ERG and ETV1 gene fusions have been detected in only a limited number of BPH and High-Grade Prostate Intra-epithelial Neoplasia (HGPIN) lesions, albeit in the absence of ETS upregulation [112,113]. In one cohort *TMPRSS2–ERG* T1/E4 fusion associated upregulation of ERG was found to be highly prognostic of disease recurrence [114] independent of grade, stage and PSA level [105]. ETS fusions are strongly linked with survival when associated with loss of PTEN (P < 0.001) [115] and have been identified in 48% of men who died of castrate-resistant disease [116]. Fusion-related upregulation of ERG is increased by oestrogens and the incidence and prognostic significance of these gene fusions may vary with cohort race/ethnicity and different techniques of detection [108,115,117,118,119,120,121]. Electrophoresis after RT-PCR using fusion specific primers followed by Southern transfer and probing with fusion specific probes appears to be a more rigorous method of detection [110].

## 7. Other Markers of Detection

### 7.1. Early Prostate Cancer Antigen

Leman *et al.* [122] reported results on a serum biomarker called early prostate cancer antigen (EPCA) using an antibody assay against the EPCA-2.22 epitope. The study involved 385 men and reported a 92% specificity for healthy men and men with benign prostatic hyperplasia and a 94% sensitivity for overall PCa detection. In addition, the authors indicated that EPCA-2.22 was highly accurate in differentiating between localized and extracapsular disease [122]. More recently, the same group reported on a second epitope of EPCA, designated EPCA-2.19, which they found provided almost identical results [123]. Despite the promising nature of these findings, more details are required including the results from multicentre studies. As a result of challenges to the work regarding EPCA-2, the place of this candidate as a PCa marker is currently uncertain [124].

### 7.2. GOLPH2

GOLPH2/GP73, elevated in PCa tissues, is detectable in post-prostatic massage urine from PCa patients. GOLPH2 immunihistochemical staining indicates a perinuclear Golgi-type pattern that is more intense in PCa glands compared with normal glands (P < 0.001) [125]. In a large study by Kristiansen *et al.* [125], upregulation of GOLPH2 protein was reported in 567 of 614 tumours (92.3%) and AMACR in 583 of 614 tumours (95%) (correlation coefficient 0.113, P = 0.005). Importantly, GOLPH2 immunohistochemical analysis indicates a lower level of intratumoral heterogeneity (25 *vs.* 45%). Further, GOLPH2 upregulation was detected in 26 of 31 (84%) AMACR-negative PCa cases. Using PCR analysis, Laxman *et al.* [26] demonstrated increased GOLPH2 levels in post prostatic massage urines as a significant predictor of PCa when multiplexed with PCA3 and SPINK1 [26].

### 7.3. SPINK1

SPINK1 (also referred to as TAT1) is a biomarker for PCa that can be detected in prostatic massage urine. SPINK1, a trypsin inhibitor secreted from pancreatic acinar cells, is thought to function in the prevention of trypsin-catalyzed premature activation of zymogens within the pancreas and the pancreatic duct. Mutations of this gene are associated with hereditary pancreatitis and tropical calcific pancreatitis [127,128]. SPINK1 is also overexpressed in other cancers, and an elevated serum level is an independent prognostic indicator in many of these, as reviewed by Paju and Paju [129,130]. Tomlins *et al.* [126] found SPINK1 expression to be an outlier (using a meta-COPA outlier meta-analysis) exclusively in a subset (10%) of ETS rearrangement-negative cancers and only in 6% of ETS negative PCa ductal adenocarcinoma variants [131]. Laxman *et al.* [26] showed that a multiplexed qPCR assay including SPINK1 on sedimented urine from patients presenting for prostate biopsy or prostatectomy outperformed serum PSA or *PCA3* alone. SPINK1 expression in urine is also an independent predictor of biochemical recurrence after resection [26,126]. The aggressive 22RV1 prostate cancer cell line expresses SPINK1 and SPINK1 knockdown attenuates 22RV1 invasion, suggesting a functional role in ETS rearrangement-negative prostate cancers [126].

### 7.4. *α*-Methylacyl Coenzyme A Racemase (AMACR)

AMACR is a very commonly used immunohistochemical marker for PCa which can also be detected in the urine of PCa patients [26,57,125]. AMACR, also known as P504S, is involved in β-oxidation of branched-chain fatty acids and fatty acid derivates. AMACR is consistently upregulated at both the mRNA and protein levels in prostate tissue [57], however, its usefulness as a biomarker in urine is controversial [26,132,133]. Western blot analysis for AMACR was used on voided urine after TRUS and biopsy, showing a 100% sensitivity and 58% specificity for PCa detection in one group of patients with negative biopsy findings [133].

## 8. Specific Prognostic Markers

As the second step in the non-invasive detection process, prognosticating is less well established with the possible exception of tissue characterisation using markers, with the presumption that the biopsied samples are representative of each gland’s malignancy status. Furthermore, although a number of markers have been reported to indicate aggressive and lethal PCa, there are considerably fewer indicators of low-risk PCa which is of particular relevance if an active surveillance or close observation clinical approach is to be pursued. 

### 8.1. AZGP1 & hCAP-D3

Zinc-alpha2-glycoprotein (AZGP1) is present in high concentration in human seminal plasma and considered to be a soluble homologue of MHC-I [135]. Hale *et al.* [136] reported that Anti-AZGP1 monoclonal antibodies reacted strongly with normal prostatic epithelium but not with other components of prostate or seminal vesicles and that 35 of 48 PCas also reacted with anti-AZGP1 antibodies. However, it was notable that high-grade tumours expressed significantly less AZGP1 than moderate-grade tumours. In addition, men with AZGP1-producing PCas had elevated levels of serum AZGP1 relative to normal age- and race-matched controls (P < 0.02). More recently, Bondar *et al.* [137] have developed a serum bio-assay for AZGP1 [137].

Henshall *et al.* [138] reported on the highly predictive capacity of AZGP1 expression in radical prostatectomy (RP) specimens. More recently, Lapointe *et al*. [139] reported a combination of immunohistology for AZGP1 and RNA *in situ* hybridisation for hCAP-D3 expression in tissues from 225 RP specimens which distinguished even more clearly those patients whose tumours would recur and those whose would not (p = 0.0002). Loss of both hCAP-D3 and AZGP1 expression was associated with the worst outcomes whereas expression of both markers indicated a very low recurrence rate raising the potential for identifying those patients better suited to an active surveillance protocol than an active treatment approach with curative intent. 

### 8.2. Prostatic Acid Phosphatase *(PAcP)*

Prior to the introduction of serum PSA, PAcP was used widely to indicate advanced PCa, but fell into disuse. However, a number of publications have promoted a renewed role for this enzyme as a prognostic indicator in early disease. Moul *et al.* [140] reported on 295 patients who underwent RP and compared the value of pre-treatment serum PSA and PAcP. The Kaplan-Meier disease-free survival rate at 4 years was 78.8% for men with a PAcP < 3 ng/mL and 38.8% for those with PAcP ≥ 3 ng/mL, which was significant overall (p < 0.001) and, when pre-treatment PSA was <10 ng/mL (p = 0.047), ≥10 ng/mL (p = 0.012). They concluded that PAcP testing added prognostic information to pre-treatment PSA values and that PAcP was an independent predictor of recurrence [140]. 

More recently, Han *et al.* [141] reviewed pre-operative PAcP levels in 1681 men who proceeded to RP and confirmed that PAcP was an independent predictor of tumour recurrence (p < 0.001). Most recently Fang *et al*. [142] examined case histories of 193 patients with clinically localized PCa, a Gleason score ≥7 and/or a PSA level of ≥10 ng/mL treated with ^103^Pd brachytherapy and supplemental external beam radiotherapy (EBRT) between 1992 and 1996. The 10-year cause-specific survival (CSS) rate for patients with a pre-treatment PAcP level < 1.5, 1.5–2.4 and ≥2.5 U/L was 93%, 87%, and 75%, respectively (P = 0.013). The 10-year CSS rate for patients with a PSA level < 10, 10–20, and >20 ng/mL was 92%, 76%, and 83%, respectively (P = 0.393). On Cox multivariate regression analysis, PAcP (hazard ratio 1.31, P < 0.0001) and Gleason score (hazard ratio 2.37, P = 0.0007) were associated with CSS. PSA was not predictive of CSS (P = 0.393) but PAcP was a stronger predictor of CSS than PSA or Gleason score these men with higher risk PCa.

## 9. Multiple Markers

In a recent study of 423 consecutive patients treated with radical prostatectomy for clinically localized PCa, pre-operative plasma levels of Endoglin, interleukin-6 (IL-6), interleukin-6 soluble receptor (IL-6sR), transforming growth factor-beta1 (TGF-beta1), urokinase plasminogen activator (uPA), urokinase plasminogen inhibitor-1 (PAI-1), urokinase plasminogen receptor (uPAR), vascular cell adhesion molecule-1 (VCAM1), and VEGF were measured using commercially available enzyme immunoassays. Plasma IL-6 (P = 0.03), IL-6sR (P < 0.001), TGF-beta1 (P = 0.005), and V-CAM1 (P = 0.01) achieved independent predictor status after adjusting for the effects of standard post-operative features. After stepwise backward variable elimination, a model relying on RP Gleason sum, IL-6sR, TGF-beta1, VCAM1, and uPA improved the predictive accuracy of the standard post-operative nomogram model by a modest 4%. The long-term follow-up of these patients will be of particular interest [143].

### 9.1. Annexin A3 (ANXA3)

ANXA3, a recently identified PCa biomarker, has an inverse relationship to PCa progression and can be detected in the urine of PCa patients [144]. ANXA3 belongs to a family of calcium and phospholipid binding proteins that are implicated in cell differentiation and migration, immunomodulation, bone formation and mineralization in PCa metastasis [145]. ANXA3 has an inverse relationship to cancer and the immunhistochemical staining in prostatic tissue correlates with disease progression, Gleason score and malignancy [144]. The presence of ANXA3 in urinary exosomes and prostasomes might be the reason for its remarkable stability in urine [144,147]. ANXA3 has been quantified by western blot in the urine samples of patients with negative DRE findings and low total PSA (2–10 ng/mL^−1^), which is the clinically relevant group facing the biopsy dilemma. Combined readouts of PSA and urinary ANXA3 gave the best results with the Area Under the Receiver Operating Curve (AUROC) of 0.82 for a total PSA range of 2–6 ng/mL^−1^, 0.83 for a total PSA range of 4–10 ng/mL^−1^ and 0.81 in all patients [147]. 

## 10. MicroRNA Profiling

MicroRNAs (miRs) are small non-coding RNA molecules with a length of 21–25 nucleotides. They are mostly located within inter-genic chromosomal regions and can be found as solitary or clustered gene units. MiRs post-transcriptionally regulate gene expression by binding to complementary sequences within the 3’UTR of mRNAs. In most cases annealing leads to a modest down regulation of gene expression, although a few upregulating miRs have been described [148]. The first 6 to 8 bases of a miR direct its targeting and so each species may bind up to thousands of mRNAs. Very recently, researchers have demonstrated that not only can miRs indirectly disable gene function by associating with mRNAs, they can silence genes directly by adding methlyl groups and it has been proposed that epigenetic silencing by DNA methylation depends on the ratio of miRNA to its target RNA [149].

Profiling of PCa cell lines and specimens has been performed for miR expression. The first dedicated report by Porkka *et al.* [150] found expression of many species and that their expression varied with malignancy and with the cell’s androgen receptor status. Follow up reports have confirmed this finding and extended the profiling of prostate-specific miRs [151]. Evidence to date suggests that many miRs with altered expression in PCa are generic to malignancy. This includes over expression of miR-21 (which is known to suppress members of the p53 network [152,153], over expression of miRs-15a and 15b, and loss of miRs 145/34c/221 and 222. The expression of these miRs appears to be androgen dependent (in part) and results from chromosomal and epigenetic alterations. Few studies have assessed the translational use of these miRs [154,155]. 

## 11. Metabonomics/Metabolomics

Metabonomics/metalolomics involves the study of metabolites in a biological sample on a global scale, with the aim of understanding its metabolic profile and correlating it with a biological, physiological or clinical state. In oncology the identification of metabolites that have been observed to be different, and characteristically so in PCa compared with non-malignant prostates, has the potential to lead to the discovery of biomarkers that are useful for diagnosis and prognosis as well as for monitoring effectiveness of therapy. It has been known for many years that citrate and its preferred prostatic cation zinc are reduced in PCa epithelial cells, reflected in gland luminal contents and prostatic fluid whereas levels of choline and spermine are increased. However, unlike choline, spermine is decreased in prostatitis, this condition causing an elevation in serum PSA which can be a confounder in the diagnosis of PCa using serum PSA as the sole biomarker [156,157,158,159,160,161].

A variety of mass spectroscopic techniques is available to provide analytical information on small molecular metabolites, proteins and other molecules. They include tandem mass spectrometry (MS/MS) [162], electron ionisation (EI), electrospray, liquid secondary ionisation mass spectroscopy (LSIMS) [163], collision-induced dissociation tandem mass spectrometry (CID/MS/MS) [164] and fast atom bombardment mass spectrometry (FAB-MS) [165]. 

However, nuclear magnetic resonance spectroscopy (NMR or MRS) [166] is the method most commonly reported. Although studies have examined the prostate by *in vivo* MRS using specially designed endo-rectal coils for this purpose, this approach has decreased in recent times with an emerging interest in perfoming the MRS analyses on prostatic fluid itself or on biopsies of prostatic tissue. A number of recent reviews cover the topic in detail [166,167,168], thus this section focuses on a number of topical contributions and on the most recent developments.

Using MRS, Lynch and Nicholson [169] examined prostatic fluid from 26 patients (10 BPH, 4 PCas and 12 controls) by prostatic massage and from ejaculate from 11 men with vasal aplasia. They reported significantly lower citrate: spermine ratios in PCa (p < 0.02) and that findings from ejaculate paralleled those obtained following prostatic massage. Also using MRS a difference in citrate levels between seminal fluid from 3 patients with PCa compared with specimens from non-PCa donors, was demonstrated by Averna *et al.* [170] who subsequently confirmed this in a study of 61 participants, of whom 16 without and 21 with PCa donated seminal fluid, and 17 without and 7 with PCa donated expressed prostatic secretions. Mean citrate levels compared with those from controls were 2.7-fold lower in patients with PCa for semen (132.2 ± 30.1 *vs*. 48.0 ± 7.9 mM, p < 0.05) and expressed prostatic secretions [171]. Similarly, Serkova *et al.* [172] reported that the concentrations of citrate, spermine and myo-inositol in human expressed prostatic secretions are age-independent markers of PCa. The median concentrations of all three metabolites were significantly decreased in PCa subjects (n = 52) when compared with healthy controls (n = 26), with citrate decreasing from 349 mM to 114 mM (p < 0.0001), spermine decreasing from 57 mM to 27 mM (p < 0.002) and myo-inositol decreasing from 21 mM to 7 mM (p < 0.0001). These three markers were highly predictive of PCa with AUROC values of 0.89 for citrate, 0.87 for myo-inositol, and 0.79 for spermine. In addition, some other metabolites such as pyroglutamate and uracil [173,174] continue to emerge as potential biomarkers to indicate the presence of PCa and its prognosis.

In recent years a number of reports have emerged that use high-resolution magic angle spinning (HR-MAS) proton NMR spectroscopy to analyse the metabolite content in intact human prostate tissue samples obtained via biopsy or following surgery. As HR-MAS MRS is non-destructive, NMR and histopathological data can in principle be obtained for the same sample [175,176]. For example, the concentration of citrate (r = 0.763, p < 0.001) and spermine (r = 0.604, p < 0.018), measured by HR-MAS MRS, has been shown to correlate with the volume percentage of normal, healthy prostatic epithelial cells assessed histologically on the same samples [175]. This is physiologically significant, as spermine is a proposed endogeneous inhibitor to PCa growth, and the findings mirror solution MRS results [173], as well as other HR-MAS changes reflecting a decrease in spermine and other polyamines in PCa. 

In two contributions, Swanson *et al.* [176,177] observed different metabolite profiles in healthy glandular and stromal tissue compared with PCa in post-surgical prostate samples using HR-MAS MRS. Healthy glandular tissue contained significantly higher levels of citrate (43.1 ± 21.2 mmol/kg) and polyamines (PA) (18.5 ± 15.6 mmol/kg), and lower levels of the choline-containing compounds choline (3.52 ± 1.44 mmol/kg), phosphocholine (PC) and glycerophosphocholine (GPC) ([t-choline]_mean _ = 7.06 ± 2.36 mmol/kg) than PCa tissue ([Citrate]_mean_ = 19.6 ± 12.7 mmol/kg, p < 0.01; [PA]_mean_ = 5.28 ± 5.44 mmol/kg, p < 0.01; [t-choline]_mean_ = 13.8 ± 7.4 mmol/kg, p < 0.01). Healthy stromal tissue contained lower levels of choline compounds ([t-choline]_mean_ = 7.04 ± 3.10 mmol/kg, p < 0.01) than PCa, but was similarly low in citrate ([Citrate]_mean_ = 16.1 ± 5.6 mmol/kg) and polyamines ([PA]_mean_ = 3.15 ± 1.81 mmol/kg) [177]. In addition, levels of taurine, myo-inositol and scyllo-inositol were higher in PCa than either healthy tissue component [176]. Remarkably, larger increases in choline and decreases in citrate and polyamines (p = 0.05) correlated with more aggressive cancers [176]. Using an improved quantitation method by combining HR-MAS MRS with T_1_ and T_2_ relaxation times optimisation and internal concentration standards, Swanson *et al.* [177] confirmed their earlier observations [176]. In addition, they observed elevated concentrations of lactate (69.8 ± 27.1 mmol/kg, p < 0.01) and alanine (12.6 ± 6.8 mmol/kg, p < 0.01) in PCa compared with healthy glandular or stromal tissue [177]. Elevated levels of lactate (1.59 ± 0.61 mmol/kg, p < 0.0001) and alanine (0.26 ± 0.07 mmol/kg, p < 0.0001) in PCa versus healthy samples ([lactate]_mean_ = 0.61 ± 0.28 mmol/kg; [Ala]_mean_ = 0.14 ± 0.06 mmol/kg) were also recently confirmed by combining HR-MAS MRS with the electronic reference to access *in vivo* concentrations (ERETIC) method [178] as external concentration standard on TRUS-guided biopsy samples [179]. Finally, HR-MAS MRS can be combined with the use of ^13^C-labeled substrates as probes to characterise the various metabolic pathways involved in PCa in greater detail [180,181].

As new biomarkers for PCa are discovered, it is highly likely that newer and more sensitive methods of detection promise to reveal even more markers in this technology-driven discipline. For example a recent study of metabolite profiles in urine, blood plasma and surgical tissue samples of benign, PCa and metastatic PCa, characterised levels of a large number of metabolites by LC-MS and GC-MS, and found that sarcosine levels were increased in PCa (p = 0.0004 in urine sediments, and p = 0.0025 in urine supernatants) and the increase correlated with the invasiveness of the cancer [181]. Indeed, the mere addition of exogenous sarcosine caused benign prostate epithelial cells to assume an invasive phenotype. 

In the same vein, a combination of different analytical techniques with different strengths that complement each other is likely make the process of biomarker discovery more robust. The successful route to future PCa biomarker discovery will involve a combination of different techniques and hinges on the integration of information gained from different tissues (urine, EPF, plasma, biopsies) different analytical techniques (MRS and MS) and even different hierarchical levels (genomics, proteomics and metabolomics).

## 12. Conclusions

It is expected that, when accurate markers for detection become established in routine clinical practice, those patients with normal marker profiles will be able to be spared TRUS biopsies with the accompanying potential for untoward effects. If these men are considered to be at-risk of developing PCa subsequently, non-invasive testing for abnormal marker profiles will be able to be repeated, as appropriate. By contrast, an abnormal detection marker profile indicating the presence of tumour would select patients for prognostic profiling and prostatic biopsies. A favourable prognostic profile would increase confidence in pursuance of an active surveillance or close observation management strategy. On the other hand, a most unfavourable prognostic profile would indicate a very high likelihood that the disease is no longer localised and is expected to be already micro-metastatic. Thus, the advent of reliable markers for detection and prognosis promises to facilitate tailored management to individuals with levels of confidence not currently available. At this point in time, it is evident that no one marker by itself is able to accurately detect all cancers with 100% sensitivity and specificity, let alone reliably predict outcome, so there is a move to include several markers to improve detection rates. Currently, this usually involves total serum PSA, amongst others, with the most commonly used molecular marker PCA3, testing for which is available commercially.
